# Zinc Oxide Nanoparticles Affect Biomass Accumulation and Photosynthesis in *Arabidopsis*

**DOI:** 10.3389/fpls.2015.01243

**Published:** 2016-01-12

**Authors:** Xiaoping Wang, Xiyu Yang, Siyu Chen, Qianqian Li, Wei Wang, Chunjiang Hou, Xiao Gao, Li Wang, Shucai Wang

**Affiliations:** Key Laboratory of Molecular Epigenetics of Ministry of Education, Northeast Normal UniversityChangchun, China

**Keywords:** nanoparticles, ZnO, biomass, chlorophylls, carotenoid, gene expression, *Arabidopsis*

## Abstract

Dramatic increase in the use of nanoparticles (NPs) in a variety of applications greatly increased the likelihood of the release of NPs into the environment. Zinc oxide nanoparticles (ZnO NPs) are among the most commonly used NPs, and it has been shown that ZnO NPs were harmful to several different plants. We report here the effects of ZnO NPs exposure on biomass accumulation and photosynthesis in *Arabidopsis*. We found that 200 and 300 mg/L ZnO NPs treatments reduced *Arabidopsis* growth by ∼20 and 80%, respectively, in comparison to the control. Pigments measurement showed that Chlorophyll a and b contents were reduced more than 50%, whereas carotenoid contents remain largely unaffected in 300 mg/L ZnO NPs treated *Arabidopsis* plants. Consistent with this, net rate of photosynthesis, leaf stomatal conductance, intercellular CO_2_ concentration and transpiration rate were all reduced more than 50% in 300 mg/L ZnO NPs treated plants. Quantitative RT-PCR results showed that expression levels of chlorophyll synthesis genes including *CHLOROPHYLL A OXYGENASE* (*CAO*), *CHLOROPHYLL SYNTHASE* (*CHLG*)*, COPPER RESPONSE DEFECT 1* (*CRD1*)*, MAGNESIUM-PROTOPORPHYRIN IX METHYLTRANSFERASE* (*CHLM*) *and MG-CHELATASE SUBUNIT D* (*CHLD*), and photosystem structure gene *PHOTOSYSTEM I SUBUNIT D-2* (*PSAD2*), *PHOTOSYSTEM I SUBUNIT E-2* (*PSAE2*)*, PHOTOSYSTEM I SUBUNIT K* (*PSAK*) and *PHOTOSYSTEM I SUBUNIT K* (*PSAN*) were reduced about five folds in 300 mg/L ZnO NPs treated plants. On the other hand, elevated expression, though to different degrees, of several carotenoids synthesis genes including *GERANYLGERANYL PYROPHOSPHATE SYNTHASE 6* (*GGPS6*)*, PHYTOENE SYNTHASE* (*PSY*) *PHYTOENE DESATURASE* (*PDS*), and *ZETA-CAROTENE DESATURASE* (*ZDS*) were observed in ZnO NPs treated plants. Taken together, these results suggest that toxicity effects of ZnO NPs observed in *Arabidopsis* was likely due to the inhibition of the expression of chlorophyll synthesis genes and photosystem structure genes, which results in the inhibition of chlorophylls biosynthesis, leading to the reduce in photosynthesis efficiency in the plants.

## Introduction

Nanoparticles (NPs), also known as particulate nanomaterials (NMs), are particle materials with at least one dimension in the nanoscale (1–100 nm). Because of their particular properties, such as small size, high surface-to-volume ratio and unique physical and chemical properties, the use of NPs in industries and a wide range of consumer products are increasing greatly (Stampoulis et al., 2009). These positive commercial advances have stimulated a rapidly increasing production of engineered NPs, which made nanotechnologies a rapidly developing field with an expectation that the annual value of nanotechnology-related products is going to reach one trillion dollars in 2015 (Roco, 2005). However, the increasing usage of NPs greatly increased the likelihood of their release into the environment, and has raised concerns about the impacts of NMs on health and the environment (Xia et al., 2009; Shvedova et al., 2010).

Studies in plants have demonstrated that at least some NPs can be uptaken (Etxeberria et al., 2006; Chen et al., 2010; Kurepa et al., 2010; Schwab et al., 2015a), transported (Liu et al., 2009; Cifuentes et al., 2010; Zhao et al., 2012; Wang et al., 2012b), and accumulated in specific subcellular locations such as cell vacuoles, nuclei and plasmodesmata (Kurepa et al., 2010; Schwab et al., 2015b), and NPs can alter plant physiological processes, and influence plant growth and development (Stampoulis et al., 2009; Ma et al., 2010; Burklew et al., 2012; García-Sánchez et al., 2015). For example, ultra small anatase TiO_2_ NPs have been shown to be able to enter into plant cells, accumulate in subcellular locations such as cell vacuoles and nuclei of root cells, and cause reorganization and elimination of microtubules, resulting in inhibition of root elongation in *Arabidopsis* (Kurepa et al., 2010; Wang et al., 2011). CuO NPs have been shown to be able to transport in maize via xylem and phloem (Wang et al., 2012b). Whereas silver NPs (Ag NPs) and zinc oxide NPs (ZnO NPs) treatment lead to increase in contents of free radicals, including reactive oxygen and nitrogen species (ROS/RNS) and hydrogen peroxide (H_2_O_2_) in duckweed (Thwala et al., 2013).

Effects of several NPs, including Ag NPs (Qian et al., 2013; Thwala et al., 2013; Geisler-Lee et al., 2014), aluminum oxide NPs (Al_2_O_3_ NPs) (Burklew et al., 2012; Riahi-Madvar et al., 2012), silicon dioxide NPs (SiO_2_ NPs) (Zhang et al., 2015), and ZnO NPs (Ma et al., 2013) have been studied in several different plant species. These experiments indicated that NPs have negative effects on several different aspects of plant growth and development including seed germination (Ma et al., 2013), root elongation (Wang et al., 2012a), biomass accumulation (Zhao et al., 2013). NPs have also been shown to be able to induce oxidative stress and alter gene expression in plants (Wang et al., 2013a).

ZnO NPs are among the most commonly used NPs in a variety of applications such as personal care, ceramics, paints, pigments, foods, batteries, and semiconductors (Ju-Nam and Lead, 2008), which increased the potential of their direct release to the environment. Thus the ecological risk of ZnO NPs is an important topic. As a matter of fact, the toxicity effects of ZnO NPs have been observed in several different plants species including *Arabidopsis* (Lee et al., 2010), buckweed (Lee et al., 2013), wheat (Du et al., 2011), dotted duckmeat (Thwala et al., 2013), cucumber (Zhao et al., 2013), rapeseed (Kouhi et al., 2014), alfalfa (Bandyopadhyay et al., 2015), and cowpea (Wang et al., 2013b). Most of the experiments show that the inhibition effects of ZnO NPs on plant growth and development are likely due to the induction of oxidative stress (Hernandez-Viezcas et al., 2011; Thwala et al., 2013; Bandyopadhyay et al., 2015). In consistent with this, transcriptome analysis in *Arabidopsis* have shown that most of the genes induced by ZnO NPs are ontology groups annotated as stress responsive, including both abiotic and biotic stimuli (Landa et al., 2012).

To further investigate the mechanisms of the toxicity effects of ZnO NPs on plant growth and development, we examined the effects of ZnO NPs on biomass accumulation and photosynthesis in *Arabidopsis*. We show that expose to ZnO NPs led to decrease in biomass accumulation in both shoots and roots, and that chlorophylls, but not carotenoid contents were decreased in plants treated ZnO NPs. Consistent with this observation, quantitative RT-PCR results showed that the expression levels of some chlorophyll synthesis genes and photosystem genes examined were decreased in response to ZnO NPs treatment.

## Materials and Methods

### Plant Materials and Growth Conditions

*Arabidopsis* (*Arabidopsis thaliana*) ecotype Columbia (Col-0) was used for the experiments. Seeds were sown directly into soil pots and kept in a growth room at 22°C with a light density (photosynthetic active radiation) of approximately 120 μmol m^-2^s^-1^, and a light/dark photoperiod of 16 h/8 h.

### ZnO NPs Treatment

Soil pots were prepared by filling the 5.5 cm × 5.5 cm pots with TS-1 white peat bedding substrate (Epagma) moistened thoroughly with suspensions containing 0, 50, 100, 200, 250, and 300 mg/L ZnO NPs with particle size <50 nm (Sigma), respectively. Substrate moistened with supernatants from 300 mg/L ZnO NPs suspensions were used as controls. The supernatants were obtained by centrifuging 300 mg/L ZnO NPs suspensions for 10 min at 4000 rpm, and filtered through 0.22 um-diameter filters. *Arabidopsis* seeds were then germinated and grown in the soil pots. After germination, extra seedlings were removed to ensure that every pot contains 12 plants. The plants were watered regularly till 4-week-old, then watered once every 2 days for two times with 50 ml suspensions containing ZnO NPs at the same concentrations, or supernatants from 300 mg/L ZnO NPs suspensions as described above.

### Measurement of Net Rate of Photosynthesis, Leaf Stomatal Conductance, Intercellular CO_2_ Concentration, and Transpiration Rate

Net rate of photosynthesis (*P_N_*), leaf stomatal conductance (*g_s_*), intercellular CO_2_ concentration (*Ci*) and transpiration rate (*E*) of the fully expanded fifth rosette leaves of 6-week-old plants were measured by using a portable open-flow, gas-exchange system (LI-6400; LICOR Biosciences, Lincoln, NE, USA) on the morning before the plants were harvested. The ambient CO_2_ concentration was 360 ± 10 μmol mol^-1^, the air temperature was 22°C, and humidity was about 50%. A total of four leaves per pot were measured, and measurements were repeated four times for each leaf.

The net rate of photosynthesis was calculated as CO_2_ uptake in micromole per square meter leaf area per second (μmol CO_2_ m^-2^s^-1^), leaf stomatal conductance as water vapor in mole per square meter leaf area per second (mol H_2_O m^-2^s^-1^), intercellular CO_2_ concentration as CO_2_ in micromole per mole intercellular air (μmol CO_2_ mol^-1^), and the transpiration rate as water loss in millimole per square meter leaf area per second (mmol H_2_O m^-2^s^-1^).

### Chlorophylls and Carotenoids Measurement

Chlorophylls and carotenoids contents were measured as described by Lichtenhaler and Wellburn (1983). Briefly, Chlorophylls and Carotenoids were extracted from rosette leaves from 6-week-old plants with 100% alcohol. Absorption of the extracts was measured using a spectrophotometer (721 TYPE, Shanghai analysis instrument co., LTD). Contents of Chlorophyll a (Chl a), Chlorophyll b (Chl b), total chlorophylls (C_T_), and Carotenoids (Car) were calculated using the formula described (Lichtenhaler and Wellburn, 1983).

### Biomass and Water Content Measurement

Six-week-old plants from each pot were harvested, washed thoroughly with tap water, and then distilled water. After removing excess water with paper towels, the roots and shoots were separated, and fresh weight (FW) was recorded. The samples were then oven-dried at 80°C for 15 min, followed by vacuum-dry at 40°C to a constant mass before dry weight (DW) was recorded. FW and DW per plant were calculated respectively, by divided the FW or DW per pot with 12. Water contents were calculated by using the formula: (FW-DW)/DW.

### RNA Isolation and Quantitative RT-PCR (qRT-PCR)

Total RNA were isolated from rosette leaves of 6-week-old *Arabidopsis* plants by using EasyPure^TM^ Plant RNA Kit (Transgene) and following the manufacturer’s protocols.

First strand cDNA was synthesized using 2 μg total RNA by Oligo(dT)-primed reverse transcription using the EazyScript First-Strand DNA Synthesis Super Mix (TransGen Biotech) by following the manufacturer’s instructions. Quantitative RT-PCR (qRT-PCR) was used to examine the expression of chlorophylls synthesis genes, carotenoids synthesis genes, and photosystem structure genes. *Arabidopsis* gene *ACTIN2* (*ACT2*) were used as a inner control for qRT-PCR. The primers used for qRT-PCR examination of *ACT2*, *MAGNESIUM-PROTOPORPHYRIN IX METHYLTRANSFERASE* (*CHLM*), *Mg-chelatase subunit D* (*CHLD*), *GERANYLGERANYL PYROPHOSPHATE SYNTHASE 6* (*GGPS6*), *PHYTOENE SYNTHASE* (*PSY*), *PHYTOENE DESATURASE* (*PDS*), *ZETA-CAROTENE DESATURASE* (*ZDS*), *PHOTOSYSTEM I SUBUNIT K* (*PSAK*), and *PHOTOSYSTEM I SUBUNIT K* (*PSAN*) have been described previously (Qin et al., 2007; Stephenson and Terry, 2008; Liu et al., 2015).

The primer pairs used for qRT-PCR examination of *CHLOROPHYLL A OXYGENASE* (*CAO*), *CHLOROPHYLL SYNTHASE* (*CHLG*), *COPPER RESPONSE DEFECT 1* (*CRD1*), *PHOTOSYSTEM I SUBUNIT D-2* (*PSAD2*) and *PHOTOSYSTEM I SUBUNIT E-2* (*PSAE2*) were listed in **Table 1**.

**Table 1:** Primers used in this study.

Primers	Sequences
*CAO*-qF	5′-AGTCCTTCTGCTTTATCTCTC-3′
*CAO*-qR	5′-TTCTCAACTAATCCACTCTCA-3′
*CHLG*-qF	5′-GAGATTTGTTGTGCGTGCGG-3′
*CHLG*-qR	5′-CCAGTGGAGGCCAAGTGACT-3′
*CRD1*-qF	5′-AAGAGGAAACTGGATAGAA-3′
*CRD1*-qR	5′-AAAGAAGTAACCAAAGGAA-3′
*PSAD2*-qF	5′-CAAACACACCATCACCAATC-3′
*PSAD2*-qR	5′-ACCTCGTACCTAAAGCCAAA-3′
*PSAE*-qF	5′-CACCACCATTGTGTCTTTCT-3′
*PSAE*-qR	5′-TTGACCTTGGATCCTCTCTT-3′


### Statistical Analysis

Statistical analysis was performed as described previously (Wang et al., 2015). Briefly, data were analyzed by one-way analysis of variance (ANOVA) using the statistical software SPSS 22.0 (IBM Inc., New York, USA), and compared by student-Newman–Keuls (*q*-test).

## Results

### ZnO NPs Affect Growth and Biomass Accumulation in *Arabidopsis*

**FIGURE 1:**
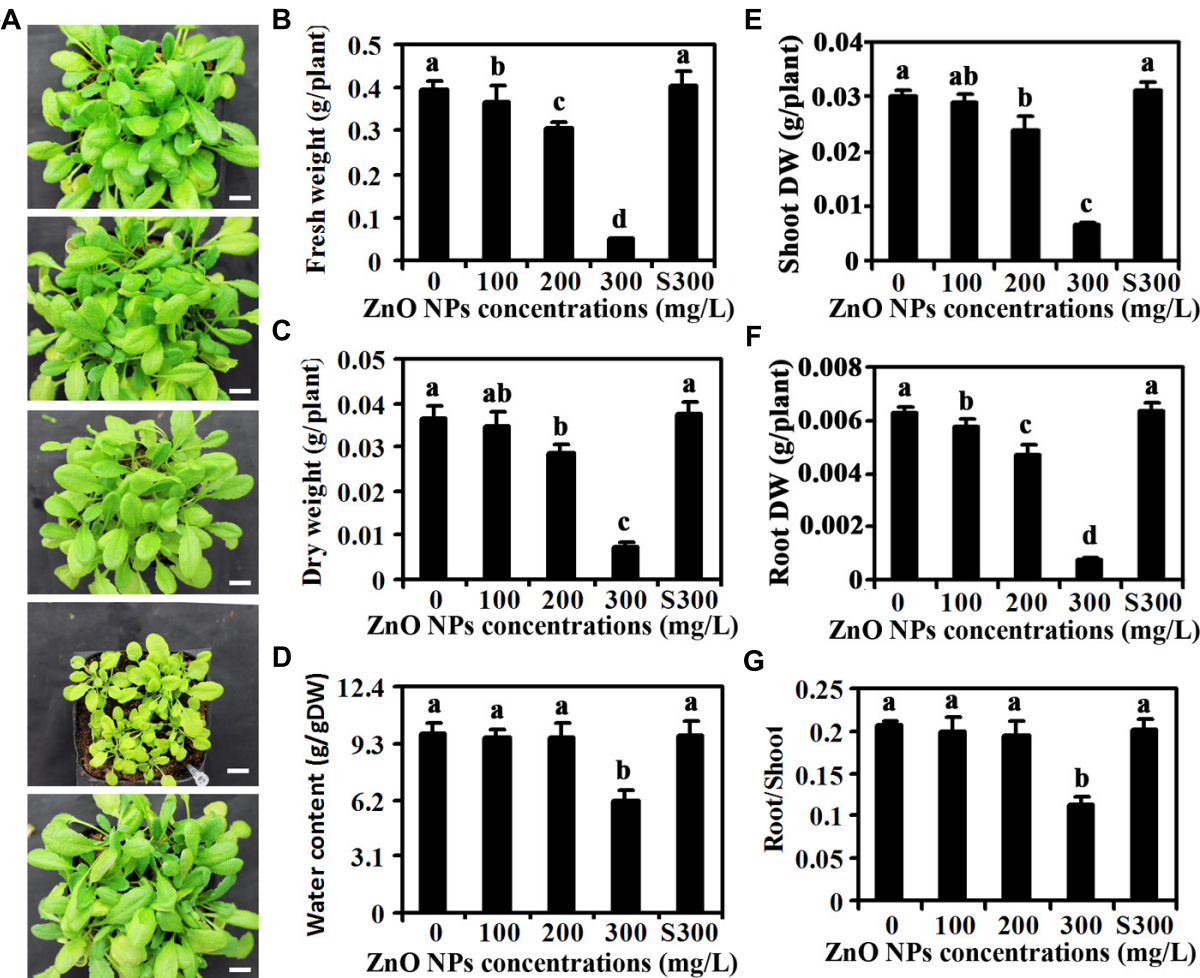
**Effects of ZnO NPs on growth of *Arabidopsis* plants.** **(A)** Photographs of 1-month-old *Arabidopsis* plants treated with 0, 100, 200, 300 mg/L ZnO NPs, or supernatant from 300 mg/L ZnO NPs suspensions (from top to bottom). Bar, 9 mm. **(B)** Fresh weight of 6-week-old *Arabidopsis* plants treated with 0, 100, 200, 300 mg/L ZnO NPs, or supernatant from 300 mg/L ZnO NPs suspensions. **(C)** Dry weight (DW) of 6-week-old *Arabidopsis* plants treated with 0, 100, 200, 300 mg/L ZnO NPs, or supernatant from 300 mg/L ZnO NPs suspensions. **(D)** Water content in 6-week-old *Arabidopsis* plants treated with 0, 100, 200, 300 mg/L ZnO NPs, or supernatant from 300 mg/L ZnO NPs suspensions. **(E)** Shoot DW of 6-week-old *Arabidopsis* plants treated with 0, 100, 200, 300 mg/L ZnO NPs, or supernatant from 300 mg/L ZnO NPs suspensions. **(F)** Root DW of 6-week-old *Arabidopsis* plants treated with 0, 100, 200, 300 mg/L ZnO NPs, or supernatant from 300 mg/L ZnO NPs suspensions. **(G)** Root/Shoot DW ratio of 6-week-old *Arabidopsis* plants treated with 0, 100, 200, 300 mg/L ZnO NPs, or supernatant from 300 mg/L ZnO NPs suspensions. Data in **(B–G)** represent the mean ± standard deviation (SD) of four replicates. Different letters indicate significantly different (*p* < 0.05).

After 1 month of growth, ZnO NPs treated plants showed an obvious decrease in the rosette size, and the decrease in the rosette sizes were positively related with the concentrations of ZnO NPs applied (**Figure 1A**), suggesting that ZnO NPs inhibited *Arabidopsis* growth. Quantitative analysis showed that ZnO NPs at 100 mg/L had little, if any effects on the FW and DW of the plants (**Figures 1B,C**), however, an about 20% of decrease on both fresh and dry weight was observed for 200 mg/L ZnO NPs treated plants, and that for 300 mg/L ZnO NPs treated plants was about 80% (**Figures 1B,C**). Water contents, on the other hand, were reduced only in 300 mg/L ZnO NPs treated plants (**Figure 1D**). Detailed analysis showed that ZnO NPs at high concentration has more severe inhibition effects on root growth than shoot growth (**Figures 1E,F**). As a result, root/shoot ratio reduced about 40% in 300 mg/L ZnO NPs treated plants, whereas ZnO NPs at relative lower concentration has little, if any effects on root/shoot ratio of the plants (**Figure 1G**).

### Chlorophylls, but not Carotenoids Contents were Affected by ZnO NPs Treatment

**FIGURE 2:**
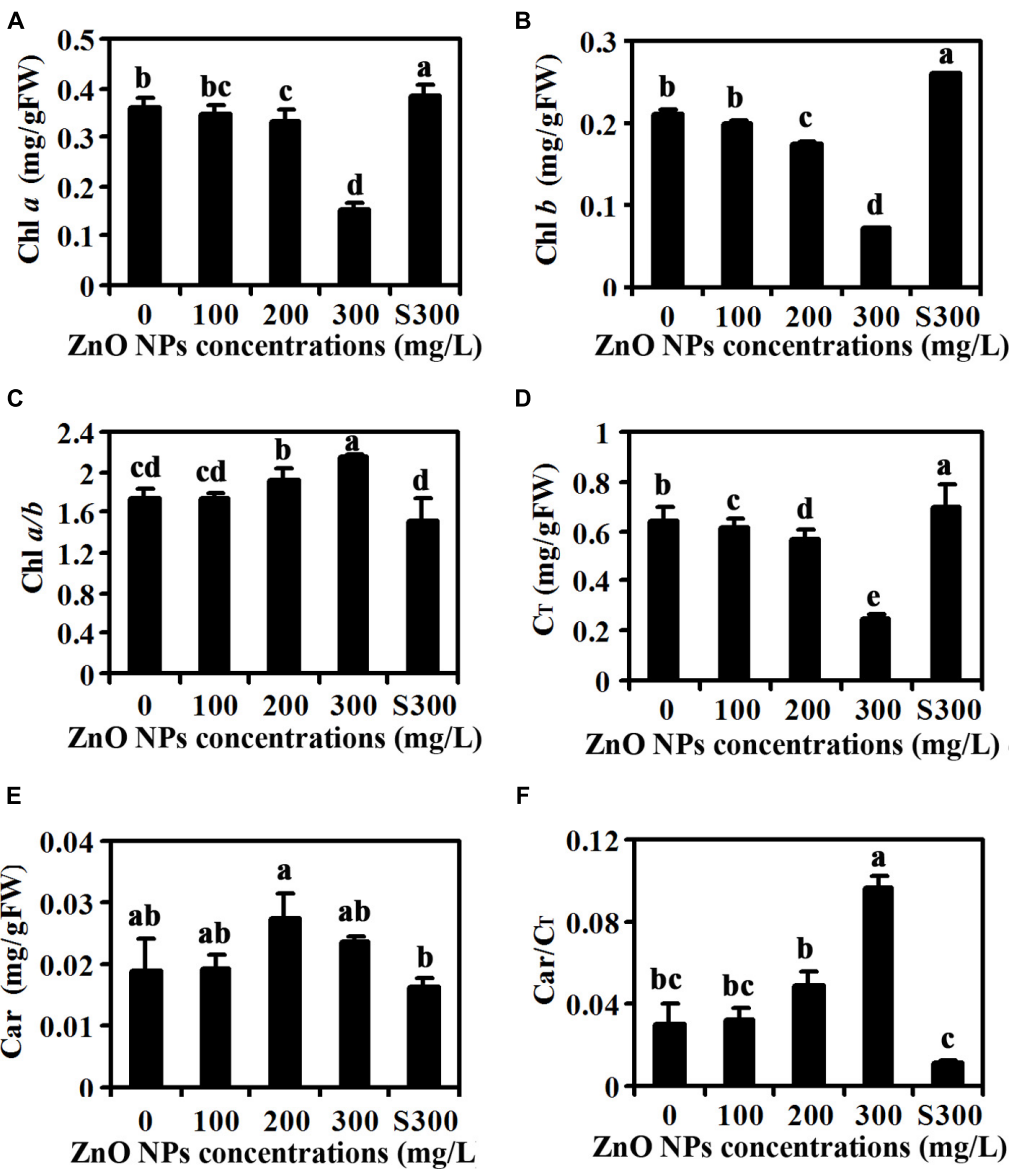
**Effects of ZnO NPs on chlorophylls and carotenoids contents in *Arabidopsis* rosette leaves.** Chlorophyll a **(A)**, Chlorophyll b **(B)**, Chlorophyll a/b ratio **(C)**, total chlorophylls **(D)**, carotenoids **(E)** and carotenoids/total chlorophylls ratio **(F)** in rosette leaves of 6-week-old *Arabidopsis* plants treated with 0, 100, 200, 300 mg/L ZnO NPs, or supernatant from 300 mg/L ZnO NPs suspensions. Chlorophylls and carotenoids were extracted from rosette leaves from 6-week-old *Arabidopsis* plants using alcohol, and measured using a spectrophotometer. Data represent the mean ± SD of four replicates. Different letters indicate significantly different (*p* < 0.05).

Yellow leaf color observed in ZnO NPs treated plants (**Figure 1A**) indicates that ZnO NPs may affect chlorophylls contents in the plants. To test this, we measured contents of chlorophylls including Chl a and Chl b in the plants. As shown in **Figure 2A**, ZnO NPs at lower concentrations has little, if any effects on Chl a contents, however, an about 50% reduce in Chl a contents was observed in 300 mg/L ZnO NPs treated plants. On the other hand, even at lower concentrations, i.e., 100 mg/L ZnO NPs treatment decreased Chl b contents in plants, and the degrees of decrease increased following the increase in the ZnO NPs concentrations (**Figure 2B**). Consistent with this, Chl a/b ratio increased, though not to a high degree in ZnO NPs treated plants (**Figure 2C**), and total chlorophylls contents also reduced in ZnO NPs treated plants (**Figure 2D**). On the other hand, ZnO NPs treatment increased carotenoids contents (**Figure 2E**). However, a positive relation between the increases of carotenoids contents and the concentrations of ZnO NPs was not observed (**Figure 2E**). As a consequence, carotenoids/chlorophylls ratio increased in ZnO NPs treated plants, and an about three-fold increase was observed in 300 mg/L ZnO NPs treated plants (**Figure 2F**).

### Leaf Photosynthesis in ZnO NPs Treated Plants was Reduced

**FIGURE 3:**
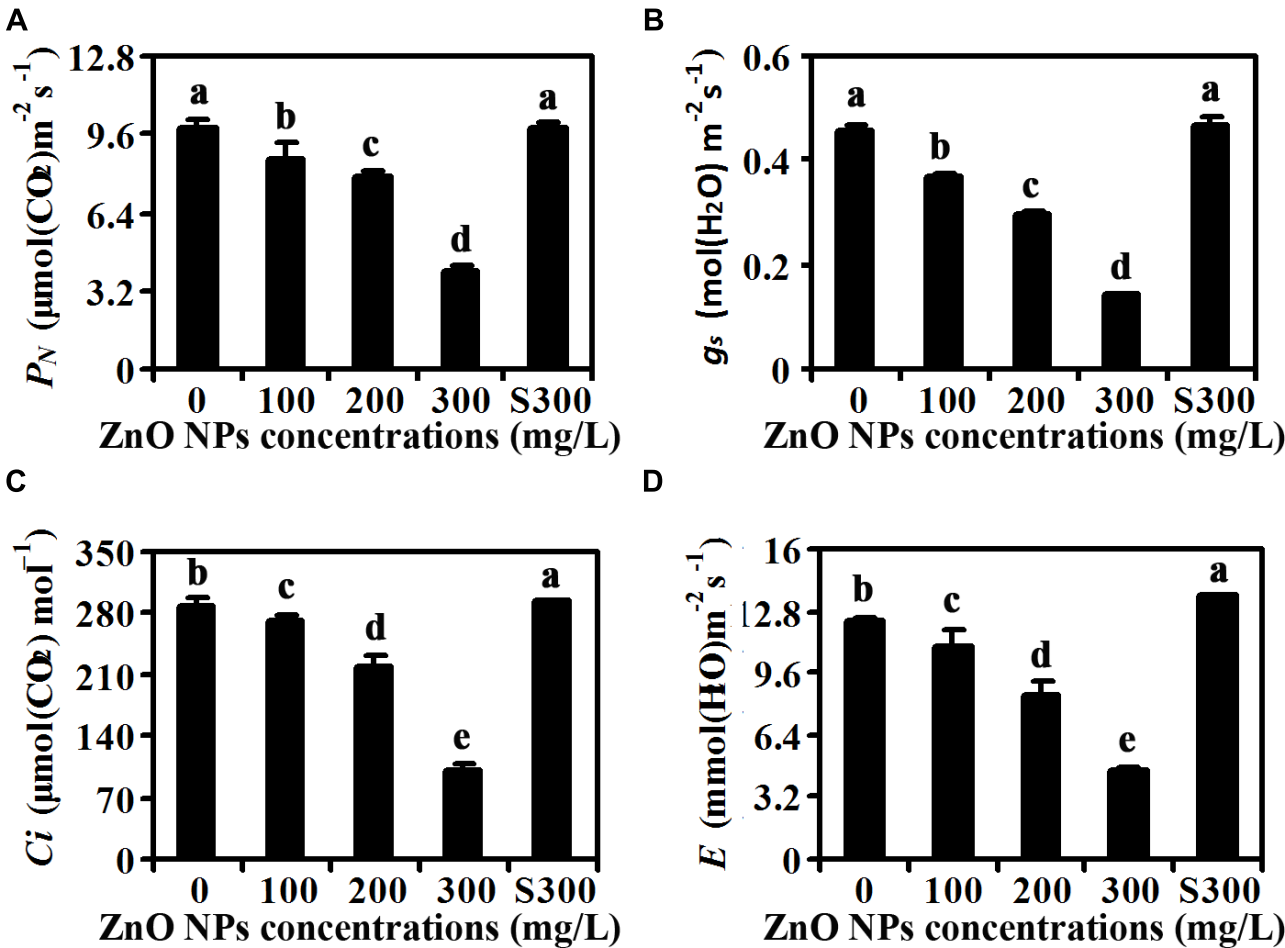
**Effects of ZnO NPs on photosynthesis of *Arabidopsis* plants.** **(A)** Net photosynthetic rate (*P_N_*), **(B)** stomatal conductance (g_s_), **(C)** intercellular CO_2_ concentration (*C_i_*), and **(D)** transpiration rate (*E*) in rosette leaves of 6-week-old *Arabidopsis* plants treated with 0, 100, 200, 300 mg/L ZnO NPs, or supernatant from 300 mg/L ZnO NPs suspensions. The fifth rosette leaves were used for the measurement by using a portable open-flow, gas-exchange system. Data represent the mean ± SD of four replicates. Different letters indicate significantly different (*p* < 0.05).

Because ZnO NPs treatment resulted in reduction of chlorophylls contents (**Figure 2**), we suspected that photosynthesis may be affected in ZnO NPs treated plants. To test this, we examined net rate of photosynthesis, leaf stomatal conductance, intercellular CO_2_ concentration and transpiration rate of the fully expanded fifth rosette leaves. As shown in **Figure 3A**, ZnO NPs treatment at all the concentrations tested decreased the net rate of photosynthesis, with an about 60% decrease observed in 300 mg/L ZnO NPs treated plants. An even higher degree of decrease was observed for the leaf stomatal conductance, in the plants treated with 300 mg/L ZnO NPs, the leaf stomatal conductance was only about 30% of that in the control plants treated with water only or with supernatants from 300 mg/L ZnO NPs suspensions (**Figure 3B**). The intercellular CO_2_ concentration was also greatly reduced in ZnO NPs treated plants, with an about 20% decrease in 200 mg/L ZnO NPs treated plants, and a more than 60% decrease in 300mg/L ZnO NPs treated plants (**Figure 3C**). A similar trend of decrease on transpiration rate was also observed in plants watered with ZnO NPs, i.e., an about 10, 30, and 60% decrease, respectively in plants treated with 100, 200, and 300 mg/L ZnO NPs (**Figure 3D**).

### Expression Levels of Chlorophyll Synthesis Genes in ZnO NPs Treated Plants were Reduced

**FIGURE 4:**
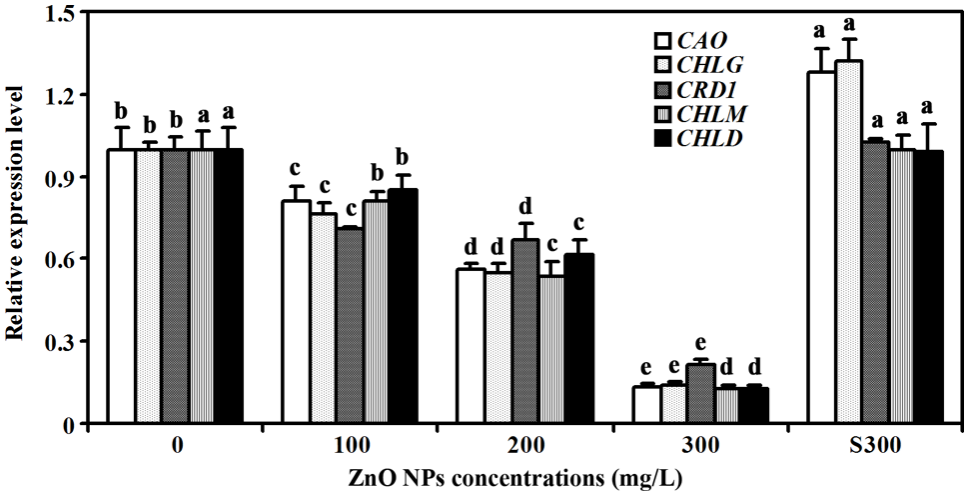
**Effects of ZnO NPs on the expression of chlorophyll synthesis genes in *Arabidopsis*.** RNA was isolated from rosette leaves of *Arabidopsis* plants treated with 0, 100, 200, 300 mg/L ZnO NPs, or supernatant from 300 mg/L ZnO NPs suspensions, and qRT-PCR was used to examine the expression of genes involved chlorophyll synthesis. Expression of *ACT2* was used as a reference gene, and expression of corresponding genes in *Arabidopsis* in the absence of ZnO NPs was set as 1. Data represent the mean ± SD of three replicates. Different letters indicate significantly different (*p* < 0.05).

Several genes including *CHLOROPHYLL A OXYGENASE* (*CAO*), *CHLOROPHYLL SYNTHASE* (*CHLG*), *COPPER RESPONSE DEFECT 1* (*CRD1*), *MAGNESIUM-PROTOPORPHYRIN IX METHYLTRANSFERASE* (*CHLM*), and *MG-CHELATASE SUBUNIT D* (*CHLD*) have been shown to be involved chlorophyll synthesis (Tanaka et al., 1998; Lange and Ghassemian, 2003; Rzeznicka et al., 2005; Pontier et al., 2007; Kobayashi et al., 2008). Having shown that ZnO NPs treatments reduced chlorophylls contents in *Arabidopsis* (**Figure 2**), we wanted to further examine if expression of the genes involved in chlorophyll synthesis were affected. Total RNA was isolated from rosette leaves, and quantitative RT-PCR was used to examine the expression of the chlorophyll synthesis genes. As shown in **Figure 4**, expression of all the genes examined including *CAO*, *CHLG*, *CRD1*, *CHLM* and *CHLD* was reduced in the plants treated with ZnO NPs at all concentrations. In 300 mg/L ZnO NPs treated plants, an ∼5–7-fold decrease of the genes examined was observed (**Figure 4**). It should be noted that a slight, i.e., ∼1.3-fold, increase for the expression of *CAO* and *CHLG* was observed in plants treated with supernatant of 300 mg/L ZnO NPs, whereas the expression of *CRD1*, *CHLM* and *CHLD* in plants treated with supernatant of 300 mg/L ZnO NPs remained largely unchanged when compared with that in water watered control plants (**Figure 4**).

### Expression Levels of Carotenoids Synthesis Genes were Increased in ZnO NPs Treated Plants

**FIGURE 5:**
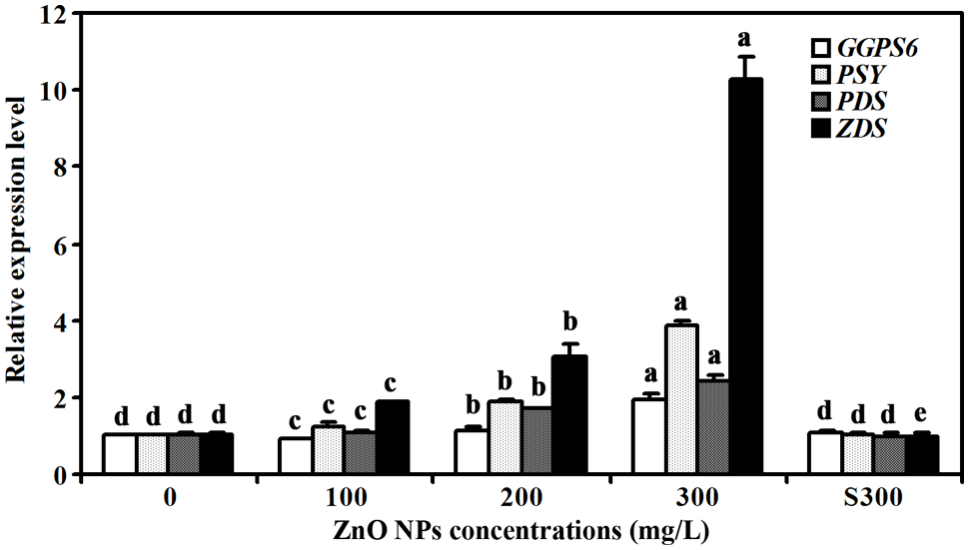
**Effects of ZnO NPs on the expression of carotenoid synthesis genes in *Arabidopsis*.** RNA was isolated from rosette leaves of *Arabidopsis* plants treated with 0, 100, 200, 300 mg/L ZnO NPs, or supernatant from 300 mg/L ZnO NPs suspensions, and qRT-PCR was used to examine the expression of genes involved carotenoid synthesis. Expression of *ACT2* was used as a reference gene, and expression of corresponding genes in *Arabidopsis* in the absence of ZnO NPs was set as 1. Data represent the mean ± SD of three replicates. Different letters indicate significantly different (*p* < 0.05).

Because carotenoids contents were increased slightly in ZnO NPs treated plants, we also examined the expression of several genes that have been reported to be involved in carotenoids synthesis, including *GERANYLGERANYL PYROPHOSPHATE SYNTHASE 6* (*GGPS6*), *PHYTOENE SYNTHASE* (*PSY*), *PHYTOENE DESATURASE* (*PDS*), and *ZETA-CAROTENE DESATURASE* (*ZDS*) (Lange and Ghassemian, 2003; Dong et al., 2007; Qin et al., 2007; Rodríguez-Villalón et al., 2009). We found that the expression of these genes was increased in response to ZnO NPs treatments, but to different degrees. In 300 mg/L ZnO NPs treated plants, an about 2-, 4-, 2- and 10- fold increase for *GGPS6*, *PSY*, *PDS* and *ZDS*, respectively was observed (**Figure 5**).

### Expression Levels of Photosystem Structure Genes in ZnO NPs Treated Plants were Reduced

**FIGURE 6:**
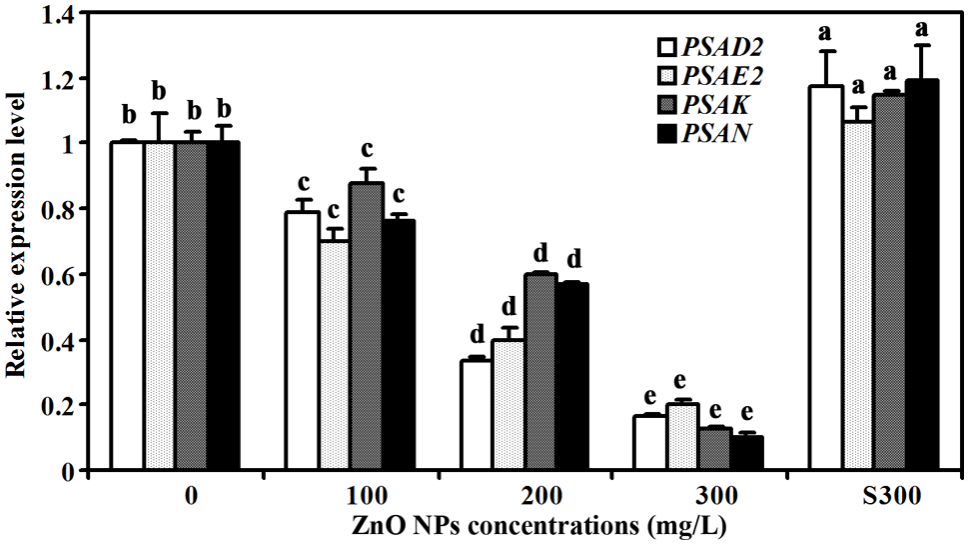
**Effects of ZnO NPs on the expression of photosystem genes in *Arabidopsis*.** RNA was isolated from rosette leaves of *Arabidopsis* plants treated with 0, 100, 200, 300 mg/L ZnO NPs, or supernatant from 300 mg/L ZnO NPs suspensions, and qRT-PCR was used to examine the expression of photosystem genes. Expression of *ACT2* was used as a reference gene, and expression of corresponding genes in *Arabidopsis* in the absence of ZnO NPs was set as 1. Data represent the mean ± SD of three replicates. Different letters indicate significantly different (*p* < 0.05).

In addition to the expression of the chlorophylls and carotenoids synthesis genes, we also examined the expression of photosystem structure genes including *PHOTOSYSTEM I SUBUNIT D-2* (*PSAD2*), *PHOTOSYSTEM I SUBUNIT E-2* (*PSAE2*), *PHOTOSYSTEM I SUBUNIT K* (*PSAK*), and *PHOTOSYSTEM I SUBUNIT K* (*PSAN*) (Varotto et al., 2002; Friso et al., 2004; Ihnatowicz et al., 2004; Peltier et al., 2004). We found that the expression of these genes was also reduced in ZnO NPs treated plants (**Figure 6**). As a control, a slight increased expression of all the four genes was observed in plants treated with supernatant of 300 mg/L ZnO NPs (**Figure 6**).

## Discussion

Previous experiments have shown that toxic effects of ZnO NPs on plants are likely caused by the induction of oxidative stress in plants (Hernandez-Viezcas et al., 2011; Thwala et al., 2013; Bandyopadhyay et al., 2015). Consistent with this, most of the genes induced by ZnO NPs in *Arabidopsis* are relative to stress responses (Landa et al., 2012). We provide evidence here in this report that ZnO NPs are toxic to *Arabidopsis* plants, and their toxic effects may due to the effects of ZnO NPs on the expression of chlorophyll synthesis and photosystem genes.

First, similar to the results obtained in other plants such as cucumber (Zhao et al., 2013), ZnO NPs treated *Arabidopsis* plants have reduced rosette size and biomass accumulation, and the reduction is positively related to the concentrations of ZnO NPs used (**Figure 1**). Our data also show that the ZnO NPs have similar inhibition effects on biomass accumulation in shoots and roots (**Figure 1**). Whereas plants treated with supernatant of 300 mg/L ZnO NPs suspensions are similar to control plants in both rosette size and biomass accumulation (**Figure 1**). These results suggest that ZnO NPs are toxic to *Arabidopsis*, though we could not rule out the possibility that the toxic effects may be partially due to the release of Zn^2+^ by ZnO NPs into the soil (Cornelis et al., 2014), it is unlikely that the toxic effects observed was due to the possible release of Zn^2+^ or other ions by ZnO NPs to the supernatants.

Second, contents of chlorophyll a and chlorophyll b were reduced in rosette leaves of ZnO NPs treated plants, whereas those in plants treated with supernatants of 300 mg/L ZnO NPs suspensions remain largely unaffected (**Figure 2**). Net rate of photosynthesis, leaf stomatal conductance, intercellular CO_2_ concentration and transpiration rate were all reduced in ZnO NPs treated plants (**Figure 3**). Carotenoids contents, on the other hand, showed slightly increase in ZnO NPs treated plants (**Figure 2**), suggesting that reduction in photosynthesis is caused by reduced chlorophylls contents. Nevertheless, these results suggest that toxic effects of ZnO NPs on *Arabidopsis* are likely caused by reduced chlorophylls contents in the plants, which in turn limited photosynthesis in the plants, leading to the reduce in biomass accumulation.

Third, consistent with the observation that chlorophylls contents were reduced in ZnO NPs treated plants, expression of chlorophyll synthesis genes was reduced in ZnO NPs treated plants (**Figure 4**). We also found the expression of photosystem genes was reduced in ZnO NPs treated plants (**Figure 6**). On the other hand, the expression of some chlorophyll synthesis and photosystem genes was slightly increased in plants treated with supernatant from 300 mg/L ZnO NPs suspensions (**Figures 4** and **6**), further confirmed that the toxic effects observed in ZnO NPs treated plants were not due to the Zn^2+^ or other ions released by ZnO NPs in to the supernatants. Though carotenoids contents only slightly increased in ZnO NPs treated plants (**Figure 2**), we found the expression of some carotenoids synthesis genes, especially *ZDS*, was dramatically increased in ZnO NPs treated plants (**Figure 5**). Considering that carotenoids is also involved in photosynthesis, and ZnO NPs are toxic to plants, it will be of great interest to find out why ZnO NPs treatment increased the expression of carotenoids synthesis genes in *Arabidopsis*.

It should be noted that when compared with a single plant or plants grown at low density, plants growth at high density showed intraspecific competition (Geisler et al., 2012; Masclaux et al., 2012; Pierik and de Wit, 2014), which led to the upregulation of photosynthesis genes (Geisler et al., 2012). However, because all the pots in our experiments contained the same number of plants, the difference of the gene expression observed was unlikely due to intraspecific competition.

Nevertheless, our results show that ZnO NPs inhibited plant growth and biomass accumulation in *Arabidopsis*, and that in addition to the induction of oxidative stresses, regulation of chlorophyll synthesis and photosystem genes expression may also contribute to the toxic effects of ZnO NPs in *Arabidopsis*.

## Conflict of Interest Statement

The authors declare that the research was conducted in the absence of any commercial or financial relationships that could be construed as a potential conflict of interest.
