# Corrigendum: Zinc Oxide Nanoparticles Affect Biomass Accumulation and Photosynthesis in *Arabidopsis*

**DOI:** 10.3389/fpls.2016.00559

**Published:** 2016-04-25

**Authors:** Xiaoping Wang, Xiyu Yang, Siyu Chen, Qianqian Li, Wei Wang, Chunjiang Hou, Xiang Gao, Li Wang, Shucai Wang

**Affiliations:** Key Laboratory of Molecular Epigenetics of MOE, Northeast Normal UniversityChangchun, China

**Keywords:** nanoparticles, ZnO, biomass, chlorophylls, carotenoid, gene expression, *Arabidopsis*

Reason for Corrigendum:

The name of the seventh author has been misspelled as “Xiao Gao,” which should be spelled as “Xiang Gao.” The authors apologize for the mistake. This error does not change the scientific conclusions of the article in any way.

## Author contributions

SW, XG, and LW conceived the study, and designed the experiments. XW, XY, SC, QL, WW, and CH performed the experiments. XW, SW, XG, and LW analyzed the data. SW drafted the manuscript, and all authors read and approved the final manuscript.

### Conflict of interest statement

The authors declare that the research was conducted in the absence of any commercial or financial relationships that could be construed as a potential conflict of interest.

